# Klinefelter syndrome as a window on the aetiology of language and communication impairments in children: the neuroligin–neurexin hypothesis

**DOI:** 10.1111/j.1651-2227.2011.02150.x

**Published:** 2011-06

**Authors:** Dorothy VM Bishop, Gaia Scerif

**Affiliations:** Department of Experimental Psychology, University of OxfordOxford, UK

**Keywords:** Autism, Klinefelter syndrome, Language impairment, Neurexin, Neuroligin, Sex chromosome trisomy

## Abstract

**Aim:**

To compare the phenotype in Klinefelter syndrome (KS) with (i) specific language impairment (SLI) and (ii) XXX and XYY trisomies.

**Methods:**

Phenotypes of KS, XXX and XYY were based on data from a systematic review of neurodevelopmental outcomes plus a recent parent survey. Phenotype of SLI was based on a published survey of children attending a special school.

**Results:**

There are close similarities between the KS phenotype and SLI. Furthermore, a minority of children with KS have features of autistic spectrum disorder. Similar language and communication problems are seen in the other two sex chromosome trisomies (SCTs), XXX and XYY.

**Conclusion:**

We propose the neurexin–neuroligin hypothesis, based on the observation that neuroligin genes, which occur on both X and Y chromosomes, are involved in the same synaptic networks as neurexin genes with common variants that affect risk for SLI and autism. According to our hypothesis, the effect of a triple dose of neuroligin gene product will be particularly detrimental when it occurs in conjunction with specific variants of neurexin genes on other chromosomes. This speculative proposal demonstrates the potential of illuminating the aetiology of common neurodevelopmental disorders by studying children with SCTs.

In this article, we focus on three aspects of the Klinefelter syndrome (KS) phenotype. The first concerns the close similarities between the phenotype in KS and that seen in specific language impairment (SLI), a behaviourally defined neurodevelopmental disorder with complex and presumed multifactorial aetiology. Second, we note that, although there are phenotypic differences between different sex chromosome trisomies (SCT), XXX and XYY share with XXY a tendency to have problems with language and communication. Insofar as there are similarities, this would seem to imply that the phenotype is affected by genes on the X chromosome that escape inactivation and that have a homologue on the Y chromosome. The final point we need to take into account is the substantial phenotypic variation within groups of children with KS and those with other SCTs. We propose that all three pieces of evidence can be explained in terms of an integrative hypothesis that postulates that interactions between genes in the same neurexin–neuroligin network are implicated in causing language and communication difficulties.

## The Neuropsychological Profile in Klinefelter Syndrome

The neuropsychological profile in XXY has many features in common with specific language impairment (SLI), a condition that is diagnosed in children of presumed normal karyotype when language development is out of step with other aspects of development (1). The diagnosis of SLI is made on the basis of cognitive/linguistic characteristics, when a child has normal nonverbal IQ but is delayed in early language development and continues to have verbal deficits in childhood and adulthood. Expressive language is typically more severely affected than receptive language, although both are usually impaired to some extent. Problems with verbal short-term memory, grammar and phonological processing are often noted, and literacy is typically poor. Although SLI is regarded as a ‘specific’ disorder, there is an association with poor motor skills (2) and attention deficit hyperactivity disorder (ADHD) (3). In most children with SLI, the aetiology is presumed to be complex and multifactorial, and no specific chromosomal or genetic abnormalities are found, though three cases of KS were found in a survey of 82 children attending a special school for children with severe speech and language impairments (4). Table 1 shows a direct comparison between SLI and the phenotype of KS. The characteristics of SLI are taken from a survey of children attending a residential school for children with specific speech and language impairments (5), and the information on KS is from the systematic review by Leggett et al. (6) of the cognitive profile in individuals identified prenatally or through newborn screening, and a recent study by Ross et al. (7).

**Table 1:** Comparison of clinical features of the phenotype of SLI and KS

Common clinical characteristics of SLI*	Seen in KS?†
Delay in early language milestones	√
Delay in starting to walk	√
Receptive language deficits but milder than expressive	√
Word finding below CA level	√
Expressive grammatical difficulties	√
Speech difficulties	√
Poor literacy skills	√
Severe limitation of verbal memory span	√
Verbal IQ much lower than Performance IQ	√
Poor attention	√
Poor peer relationships/solitariness	x
Motor clumsiness, especially for gross motor skills	√

SLI = specific language impairment; KS = Klinefelter syndrome.

* Features reported in at least 30% of cases in the survey by Haynes and Naidoo (5) of children attending a residential school for children with specific speech and language impairment.

† Based on Leggett et al. (6), and Ross et al. (7).

It can be seen that there are clear parallels between SLI and KS for nearly all of the characteristics shown in Table 1. In agreement with this, our recent study of prenatally diagnosed children with KS (10) found that nine of 19 (47%) children with KS had received speech and language therapy. Parents also reported poor communication and social relationships on quantitative scales (8). Attentional deficits on neuropsychological measures were also noted in a large-scale study of children with KS recruited from an endocrinological clinic (9). In our sample (10), consistent with research by van Rijn and colleagues (11), parents reported that 2/19 (11%) of KS cases had received a diagnosis of autistic spectrum disorder (ASD) by a professional, a diagnosis that can be used to refer to core autistic disorder, but is also applied to children with milder features of autism. This is consistent with a self-selected sample of Dutch boys with KS (12), which found 65% met criteria for language disorder, 63% had attention deficit disorder and 27% had an autism spectrum disorder.

This latter observation raises questions about the relationship between SLI and ASD. Although these conditions are generally regarded as quite distinct, the boundaries between them can be hard to draw (13), and it has been suggested that there is some genetic overlap, with variants of the CNTNAP2 neurexin gene being associated with both SLI and autism (14).

## Overview of Phenotypic Similarities and Differences Among KS, XYY and XXX

One difficulty in studying XXX and XYY trisomies is that, unlike KS, there is no impact on sexual development, and therefore because the neurocognitive phenotype is often mild, many cases go undetected. Individuals whose trisomy is discovered during medical investigations may not be representative, especially if the investigation is for neurodevelopmental disorders. This potential ascertainment bias can be avoided by studying outcomes of children with SCTs discovered on neonatal screening, several of which were initiated in the 1960s. Results from these studies were integrated in the systematic review by Leggett et al. (6), which we draw on heavily in the comparisons below. No new studies of this kind have been initiated since that time. It is, however, increasingly common to find cases identified on prenatal screening, and we also refer to findings for the subset of children identified this way who were included in the study by Bishop et al. (10).

### Comparison between KS and XXX

One striking similarity between KS and XXX cases is that language is impaired. Bishop et al. (10) found that 7 of 30 (24%) of girls with XXX had received speech and language therapy. Furthermore, motor impairments have been independently noted for girls with XXX (15) and boys with KS (16). In previously unpublished data from our sample, parents reported fine and gross motor control as equally problematic for prenatally diagnosed children with XXX and XXY, with an approximate threefold increase in difficulties compared to their brothers or sisters (see Table 2). There are, however, some key differences between the phenotypes of KS and XXX. First, the intellectual profile is more uneven in KS; as with SLI, these boys tend to have higher nonverbal than verbal abilities, whereas in girls with XXX, both verbal and nonverbal skills are impaired to an equal extent (6). Second, autism spectrum disorder was not found in any of the XXX girls in our 2010 study, and on a communication checklist, although overall scores were depressed, pragmatic skills showed less impairment than for boys with KS (10).

**Table 2:** Parental report of motor difficulties by karyotype. Mean (SD) raw scores, with high scores indexing greater difficulties. Fine motor max score = 24; Gross motor max score = 48; Total motor max score = 72. See Data S1 for details of checklist.

		Fine motor		Gross motor		Total difficulties	
				
	N	Mean	SD	Mean	SD	Mean	SD
XXX	28	6.9***	4.9	9.0***	7.5	15.9***	11.5
XXY	19	8.1**	5.6	8.2**	7.2	16.3***	11.6
XYY	21	9.1***	5.9	14.4***	11.0	23.5***	15.9
Siblings							
XX	26	2.2	3.7	2.1	2.4	4.3	5.8
XY	39	2.9	3.4	3.4	5.5	6.3	8.5

* Scores are poorer than same-sex siblings

** p < 0.005

*** p < 0.001

Mann–Whitney *U*.

### Comparison between KS and XYY

Mild deficits in IQ, especially VIQ, are seen in boys with XYY as well as in KS (6). Furthermore, our study using parental report (10) found a suggestion of especially high rates of language difficulties in boys with XYY, with 15 of 21 (71%) of cases having a history of speech and language therapy. Boys with KS and XYY showed remarkably similar profiles of impairment on a communication checklist, and both had an elevated risk of diagnosis of ASD, with this diagnosis being reported for 4 of 21 (20%) of the XYY cases. Just like boys with KS, boys with XYY experience difficulties with aspects of attentional and executive control (7) and both fine and gross motor difficulties; see Table 2 and (7).

## Why is Language/Communication Affected in all Three SCTs?

The relatively mild impact of trisomy of sex chromosomes compared to autosomes can be explained in terms of two factors. The first is that most genes on the Y chromosome are involved in sex-specific characteristics: an additional Y chromosome in men with XYY karyotype has relatively little impact because the Y chromosome contains so few genes that affect cognition. The second is X-chromosome inactivation: for women with XX or XXX karyotype and men with XXY karyotype, this ensures that only one X chromosome is fully functional; methylation affects additional X chromosomes so that the majority of genes are inactivated (17). If X inactivation were complete, we might expect to see no phenotypic differences between individuals with SCTs and those with the usual complement of 46 chromosomes. There is, however, a region in X and Y chromosomes, the pseudoautosomal region, that functions like an autosome, with homologous genes being expressed from both X and Y. Furthermore, around 15% of genes outside the pseudoautosomal region escape inactivation to some extent (18). Genes that escape inactivation have excess transcription product in individuals with SCTs.

To explain the similarities in language phenotypes between the three SCTs, we need to identify genes on the X chromosome that escape inactivation and that have a homologue on the Y chromosome. The neuroligin genes emerge as strong candidates. There are two NLGN genes on the X chromosome: NLGN3 and NLGN4X. The latter is located on Xp22 where the majority of genes are expressed from both the active and the inactive X (19). Furthermore, a homologue of NLGN4X, NLGN4Y, is located on the male-specific region of the Y (20) and is expressed in brain. Neuroligins are cell adhesion molecules that are implicated in regulating synaptic plasticity and have been posited as playing a role in neurodevelopmental disorders, especially those involving language and social interaction (21). Both under- and over-expression of these genes could disrupt functioning of circuits important for neural homeostasis. Further, mutations of neuroligin genes have been found in cases of autism (22). Nevertheless, it is not straightforward to explain autistic-like behaviours in SCTs in terms of excess dosage of neuroligin. Some studies have failed to find neuroligin mutations in ASD samples (23) and conversely, individuals have been described who have no evidence of ASD or language impairment despite a deletion affecting neuroligin (24). Furthermore, two studies of common polymorphisms of X-linked neuroligins in ASD found only limited evidence of association (25,26). This suggests, then, that mutation or duplication of neuroligins acts as a risk factor for ASD, but that risk only becomes manifest when other risk factors are present.

## Individual Differences Within Groups of Children with SCTs

Any account of phenotypes in children with SCTs has to explain not only the typical phenotype but also the variable outcomes seen in children with an extra sex chromosome. Although we have emphasized the high rates of language and communication problems in children with SCTs, we also noted that, in a group recruited via prenatal diagnosis, 37% of KS, 55% of XXX and 14% of XYY cases had no evidence of neurodevelopmental or educational difficulties (10).

There are numerous possible explanations for the variable phenotypes seen in SCTs (27). The phenotype may depend on the specific alleles that are present in treble dosage – for example, if an individual with an extra X chromosome is homozygous for a recessive allele associated with impairment, then an adverse phenotype may be manifest, whereas if there is heterozygosity, then there may be no impairment. Other genetic factors that could account for individual differences in people with SCTs are imprinting – i.e. whether an extra X chromosome (in XXX or KS) is inherited from the mother or father – variability in rates of X inactivation and mosaicism. The impact of a SCT could also depend on environmental factors (28). While acknowledging these possible explanations for phenotypic variation, we focus here a more specific hypothesis that is suggested by the marked similarities between SLI and KS, the neurexin–neuroligin (NN) hypothesis.

## The Neurexin–Neuroligin (NN) Hypothesis

The NN hypothesis maintains that language impairment arises when there is impairment in a network important for synaptic signalling involving neurexins and neuroligins. This hypothesis is in part inspired by the proposal by Ramocki and Zoghbi (21) that gene over- or under-expression will interfere with homeostasis in neuronal networks, which is needed to allow neurons to continue to carry out routine functions during development, at the same time as changes are occurring in neurogenesis and synaptic connectivity. Both neuroligin and neurexin genes are important in this regard because their interaction plays a key role in the regulation of dendritic spines and synapse structure. Together they form a cell adhesion complex that is dynamically regulated to alter cell structure and function. The NN hypothesis maintains that over-expression of neuroligins in children with SCTs has especially severe consequences when it occurs in conjunction with common variants of neurexins that are already identified as risk factors for both SLI and autism.

As noted above, a neurexin gene on chromosome 7, CNTNAP2, has been associated with a range of neurodevelopmental disorders, including SLI and autism. Variants of CNTNAP2 have been associated with a measure of phonological short-term memory in a probands with SLI and their relatives (14) and with timing of early language milestones in an autistic sample (29). There are two important points to note about these findings: first, the risk variants of CNTNAP2 are relatively common; in the SLI families studied by Vernes et al., 12% of probands had two copies of the risk haplotype, and 44% had one copy. Further, the effect size of the risk variant is modest (0.4 SD). It follows that CNTNAP2 variation is not ‘the cause’ of SLI or autism, but rather acts as a risk factor that leads to clinically significant impairment only when it occurs in conjunction with other risk factors. Using a series of simulations, Bishop (13) argued that CNTNAP2 may confer risk for SLI, but may also lead to autism only when there is a specific conjunction of a risk haplotype on CNTNAP2 with an autism risk haplotype on another gene. The NN hypothesis proposes a similar mechanism: the increase in neuroligin gene product may have a detrimental impact on language development only when the risk variant of CNTNAP2 is present, leading to a ‘double hit’ on the network involved in synaptic regulation. This hypothesis is highly speculative, but it generates testable predictions about causes of individual variation in language and communication in individuals with SCTs. For instance, we predict that a risk allele in CNTNAP2 that has only a modest effect in children of normal karyotype will have its effect magnified in those with an SCT. Thus, genetic variants of CNTNAP2 might predict which individuals with SCT have SLI and/or autism. Conversely, if these findings are confirmed in SCTs, this could lead to identification of other autosomal genes in the same functional circuits that would be predicted to play a role in SLI or autism, especially when occurring in combination with other risk factors. We have explored these ideas in relation to disorders affecting language and communication, but the ‘double hit’ logic could potentially be extended to other common neurodevelopmental disorders for which presence of an SCT confers increased risk, such as ADHD and developmental co-ordination disorder.

## Concluding Comments

The neurodevelopmental consequences of SCTs are well worth studying in their own right; it is important for parents, genetic counsellors and paediatricians to be aware of the range of outcomes that are associated with having an additional sex chromosome. We suggest that the study of SCTs may, in addition, have a broader importance. They have the potential to illuminate the genetic mechanisms leading to language and communication difficulties and so may help us understand the aetiology of common conditions such as SLI and ASD.

## Appendix: Discussion Following Dorothy Bishop's Presentation

### Neurodevelopmental outcomes in a prenatally identified sample of children with sex chromosome trisomies

#### Ronald Swerdloff (Los Angeles, USA)

You present a vast amount of fascinating data from these individuals and much more investigation is required. Can you explain why the abnormalities in cognitive function in the two groups of patients are of similar character but of greater severity in the XYY patients in comparison to the XXY patients? It appears rather surprising that the manifestations are greater in the XYY patients who have normal androgen levels whereas the XXY patients have low levels of androgen. Can this help to explain the role of androgen deficiency in Klinefelter syndrome (KS) patients?

#### Dorothy Bishop

It is possible that low androgen levels give protection from the adverse effects of extra chromosomal material. The presence of high testosterone in combination with a specific genotype may accentuate the risk. We have submitted a proposal to extend these investigations and hope to receive funding.

#### Niels E Skakkebæk (Copenhagen, Denmark)

We have seen 47,XXY and 47,XYY kids both in the clinic and prenatally, and some of the reported differences may be due to the KS children being better able to hide their symptoms. They tend to be subordinate and do as their teacher tells them whereas XYY children are all over the place. This may make their condition more obvious resulting in a request for help.

#### Dorothy Bishop

That concept can also be applied to the XXX girls who may have disabilities which are not brought to light. We know that reading difficulties and dyslexia in girls are more prevalent from epidemiological screens of populations than in children seen in the clinic. This may be because girls sit quietly at the back of the class and are unnoticed whereas boys are more active and obvious. These children should be seen and assessed.

#### Carole Samango-Sprouse (Davidsonville, USA)

Who made the diagnosis of autism in your subjects?

#### Dorothy Bishop

That is an impossible question to answer. The children were referred with a diagnosis of autism probably made by a clinician using non-precise methods.

## SUPPORTING INFORMATION

**Data S1** Materials for assessment of motor function.
