# Supplementary material for: Klinefelter syndrome as a window on the aetiology of language and communication impairments in children: the neuroligin–neurexin hypothesis
Source: Acta Paediatr. 2011 Jun;100(6):903–7. doi: 10.1111/j.1651-2227.2011.02150.x (PMC3107947; doi:10.1111/j.1651-2227.2011.02150.x)
Supplement: Supplementary file 1 [file apa0100-0903-SD1.doc]

**Supporting information**

We adapted the Movement ABC – 2, Checklist Version (Henderson & Sugden, 2007), a questionnaire tapping motor development and appropriate for children up to 12 years of age. Each item was rated as ‘fine’ (0), 'just ok' (1), ‘finds hard’ (2), or ‘no opportunity to observe’ (omitted from total). Some items were added or modified to make the scale appropriate for children up to 16 years of age, as follows (F denotes fine motor, and G gross motor):

• Ties shoe laces (F)

• Uses a knife without a fork (e.g. to cut bread, whilst holding the loaf with the other hand)

(F)

• Uses both a knife and fork together (F)

• Writes neatly and quickly (F)

• Can carry out detailed craft work (e.g., model making, drawing) (F)

• Is able to put a key in a lock at first attempt (F)

• Can peel fruit/potatoes (F)

•  Can hammer in a nail (F)

•  Puts on socks while standing up (G)

•  Runs smoothly and without tripping (G)

•  If they participate in dodging and chasing games, how do they get on? (G)

•  If they play in team sports, how do they get on? (G)

•  Can skip using a skipping rope (G)

Reference

Henderson SE, Sugden DA. Movement Assessment Battery for Children - Second Edition

(Movement ABC-2) , Checklist Version. London: Harcourt Assessment; 2007.
